# Interaction between Mean Arterial Pressure and HbA1c in Prediction of Cardiovascular Disease Hospitalisation: A Population-Based Case-Control Study

**DOI:** 10.1155/2016/8714745

**Published:** 2016-06-13

**Authors:** Dahai Yu, Zhanzheng Zhao, David Simmons

**Affiliations:** ^1^Department of Nephrology, The First Affiliated Hospital, Zhengzhou University, Zhengzhou 450052, China; ^2^Arthritis Research UK Primary Care Centre, Research Institute for Primary Care & Health Sciences, Keele University, Keele ST5 5BG, UK; ^3^Western Sydney University, Campbelltown, Sydney, NSW 2751, Australia

## Abstract

*Objective*. To explore the relationship between mean arterial pressure (MAP), HbA1c, and cardiovascular (CV) hospitalisation risk in type 2 diabetes.* Design*. Population-based case-control study.* Settings*. Primary and secondary care level in Cambridgeshire, United Kingdom.* Participants*. 588 patients with type 2 diabetes from 18 English general practices recording a CV hospitalisation in 2009–2011 were included. Risk-set sampling was used to select 2920 gender, age, and practice matched control type 2 diabetes patients.* Main Outcome Measure*. Conditional logistic regression was used to explore further dose-response relationships between MAP, HbA1c, and CV hospitalisation risk.* Results*. The relationship between MAP and CV hospitalisation was nonlinear (*P* < 0.001 for linearity test). The MAP associated with the lowest CV hospitalisation risk was 97 (95% CI: 93–101) mmHg. An interaction between MAP and HbA1c for increased risk of cardiovascular hospitalisation was observed among those with HbA1c < 7% (53 mmol/mol) and MAP < 97 mmHg.* Conclusions*. In type 2 diabetes, MAP is a good predictor of CV hospitalisation risk. CV hospitalisation is lowest with a MAP between 93 and 101 mmHg. CV hospitalisation was particularly high among those with both a low MAP and a lower HbA1c.

## 1. Introduction

Blood pressure is a major contributory factor in cardiovascular disease (CVD) [1], with systolic blood pressure (SBP) currently considered the best general population CVD risk factor [2]. Type 2 diabetes is another major risk factor for CVD [3] and its coexistence with hypertension increases CVD risk further, although this additional risk is not observed in all studies [4]. It was estimated that in the United States 30% of inpatient cost (around 22,254 million US dollars) was due to CVD hospitalisation among people with type 2 diabetes in 2012 [5]. An even greater diabetes-attributable hospitalisation cost of 46.5% was found in a major hospital in Cambridgeshire, England [6]. A paradoxical finding is that the well-known relationship between CVD and SBP in the general population was insignificant in a meta-analysis using data from people with diabetes [4]. Greater use of antihypertensive medicine and a higher prevalence of heart failure than people without diabetes have been proposed as possible reasons for this paradox [7, 8].

Mean arterial pressure (MAP) reflects both peripheral resistance and cardiac output. Recently, in the ADVANCE study [9], a trial among type 2 diabetes patients, MAP correlated with major CVD events: with a 13% increase in risk per 13 mmHg increase in MAP. If MAP is a marker for CVD risk among type 2 diabetes patients, it should be associated with greater CVD hospitalisation. However, the association between MAP and hospitalisation in type 2 diabetes has not been investigated, and a dose-response relationship between CVD hospitalisation and MAP may exist. Moreover, blood pressure and CVD are influenced by long term glycaemic control [10], usually assessed using HbA1c, which also has an association with hospitalisation risk [11].

We therefore hypothesized that MAP would predict CVD hospitalisation in type 2 diabetes in a nonlinear relationship, with an interaction between HbA1c and MAP. We also investigated whether a threshold exists below which MAP no longer predicts CVD hospital admission.

## 2. Methods

### 2.1. Settings

Secondary Uses Service (SUS) hospitalisation data up to 2010-2011 was linked with data from all patients with type 2 diabetes from 18 volunteer Cambridgeshire general practices in 2008/9 as previously described [11]. Only practices using the EMIS (Egton Medical Information Systems) GP electronic records were involved as the system allowed extraction of a predefined dataset. SUS data includes all NHS and private hospital admissions from both within and outside Cambridgeshire. All analyses were conducted on anonymised datasets and the work was approved by the Cambridgeshire Research Ethics Committee [11].

Type 2 diabetes was based upon GP recorded diagnosis, the ICD coding in the SUS dataset, current age, and the youngest recorded age at diagnosis (where both type 2 and type 1 diabetes were defined in different datasets). Hospital ICD coding (E10–E14) occurred for all patients with diabetes.

Data from general practice included demographics, body mass index (BMI), some medications, and metabolic variables including lipid profiles and HbA1c. SBP and DBP were measured over the baseline time period: 89.3% people within and 10.7% beyond the first 12 months. All blood pressure was taken before April 1, 2009, with the most recent (at least 50 days before the first admission) used in the analysis. MAP was calculated as [(2 × DBP) + SBP]/3.

Time since diabetes registration duration usually reflected the diabetes duration. Aspirin and lipid-lowering, but not diabetes, therapy were recorded along with previous CVD history.

### 2.2. Study Participants

Patients with a first recorded inpatient diagnosis of CVD as the primary code (ICD-10: I20–I25, I60–I69, and I73 in first ICD field) for the hospital admission over the 2-year time period (1 April 2009–31 March 2011) were defined as cases. The first hospitalisation date was taken as the index date for cases. Randomly sampled matched (by age, gender, and practice) controls were selected (1 : 5 case : control) using risk-set sampling [12]. Controls were assigned an index date identical to that of corresponding cases. Eligible controls had no CVD hospitalisation by the age of the matched cases.

### 2.3. Statistical Analysis

Conditional logistic regression was used for categorical variables comparisons and mixed effect models for continuous variables after age adjustment. Conditional logistic regression was used to compute odds ratios for CVD hospitalisation by mean arterial pressure levels [13]. The odds ratios estimated the incidence rate ratios as risk-set sampling of controls was used [13].

Analyses were adjusted for BMI, triglyceride, total cholesterol, low density lipoprotein, high density lipoprotein, duration of diabetes registration in GP practices, lipid-lowering and aspirin treatment, and prevalent recorded history of CVD/cerebrovascular disease.

The interaction between HbA1c and MAP in predicting hospital admission was analysed by dichotomising the relationship between two levels of HbA1c, set at the ADA usual target for HbA1c (HbA1c < 7% (53 mmol/mol) and HbA1c ≥ 7% (53 mmol/mol)).

The relationships between MAP and hospital admissions were estimated using a linear model, natural cubic spline model with 3 equally spaced knots determined from the levels of MAP, and quadratic spline model, respectively. The natural cubic spline model was chosen as the best fit model for the relationship curve by its minimum Akaike Information Criteria (AIC) compared with linear model or quadratic spline model [14]. The natural cubic spline models for the overall dataset were repeated using other potential knots, chosen to lie within the range for minimum to maximum measure of MAP (as a sensitivity analysis). The linearity of the relationship was tested using the linear test in the natural cubic spline model. The breakpoint test was carried out to target the potential thresholds (P_5_ to P_95_ of MAP measures) by incorporating the piecewise term into the cubic spline model [15]. The threshold with significant break in the regression coefficients and achieving the minimum AIC was chosen as the final threshold. The 95% confidence interval of the threshold was obtained from 1000 bootstrap samples.

The model discrimination was estimated by C-statistics (equal to the area under the Receiver Operating Characteristic (ROC) curves) from multilevel mixed effects logistic regression (two-level model), within which the matched risk-set was fixed. Mean variance inflation factor (VIF) was evaluated to avoid the impacts of multicollinearity between different variables in the model.

Two sensitivity analyses were undertaken firstly analysing the MAP measurement data rich range (covering > 95% people): 60–120 mmHg, and secondly limiting the period to CVD hospitalisation after the first three months following the start of follow-up.

All analyses were performed with STATA (STATA/SE 11.0 StataCorp, Texas). All *P* values were calculated using two-tailed tests and *P* < 0.05 was taken as significant.

## 3. Results

The 588 cases and 2920 population controls are described in Table 1, which shows the high rate of previously diagnosed coronary heart disease (66.8% versus 35.6% of controls) and higher BMI, SBP, DBP, HbA1c, low density lipoprotein, and triglyceride in cases. The average MAP was 94.1 ± 10.2 mmHg among cases and 91.3 ± 9.2 mmHg among controls (*P* = 0.0130).

**Figure 1:**
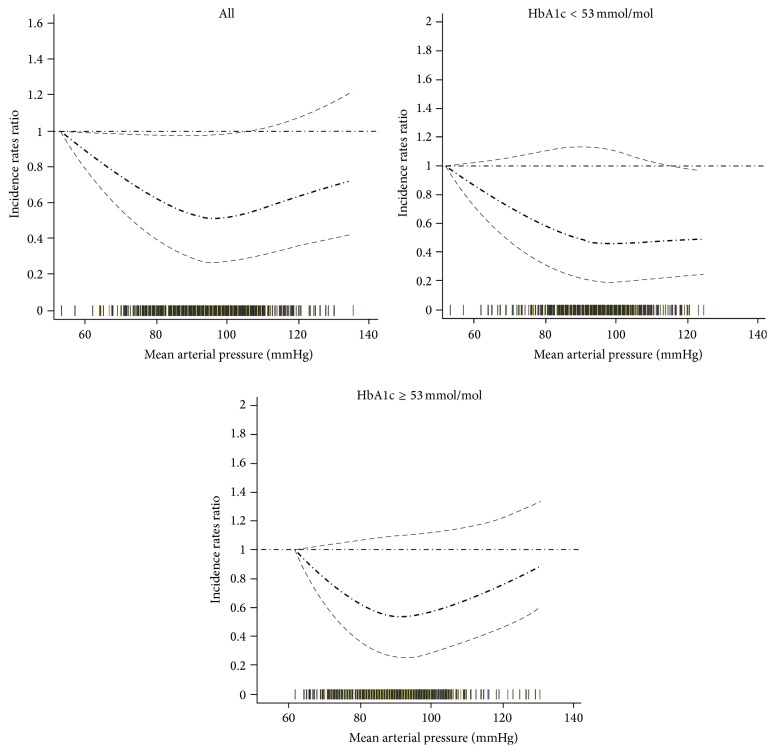
Relationship between mean arterial pressure and cardiovascular hospital admission: analysis in full data range. Adjusted for prevalent recorded history of cardiovascular/cerebrovascular disease, duration of diabetes registration in GP practices, body mass index, triglyceride, total cholesterol, low density lipoprotein, high density lipoprotein, estimated glomerular filtration rate, smoking status, lipid-lowering treatment, aspirin treatment, and pulse pressure. The thick dash-dot line indicates the incidence rate ratio and the thin dash line indicates the 95% confidence interval. The rag plot (bars on the *x*-axis) presents the mean arterial pressure distribution.

A nonlinear relationship between MAP and risk of CVD hospital admission was found (Figure 1: linearity test: *P* < 0.001), with the lowest risk of CVD hospitalisation with MAP of 97 (95% CI: 93 to 101) mmHg. Increased hospitalisation occurred above and below this MAP threshold.

An interaction between MAP and HbA1c in predicting the risk of CVD hospitalisation was observed: below the MAP threshold, people with a low HbA1c (HbA1c < 7% (53 mmol/mol)) were more likely to have a cardiovascular hospitalisation compared with those with a high HbA1c (HbA1c ≥ 7% (53 mmol/mol)). Above the MAP threshold, the risk of cardiovascular hospitalisation increased with each increment in MAP among people with a high, but not a low, HbA1c.

**Figure 2:**
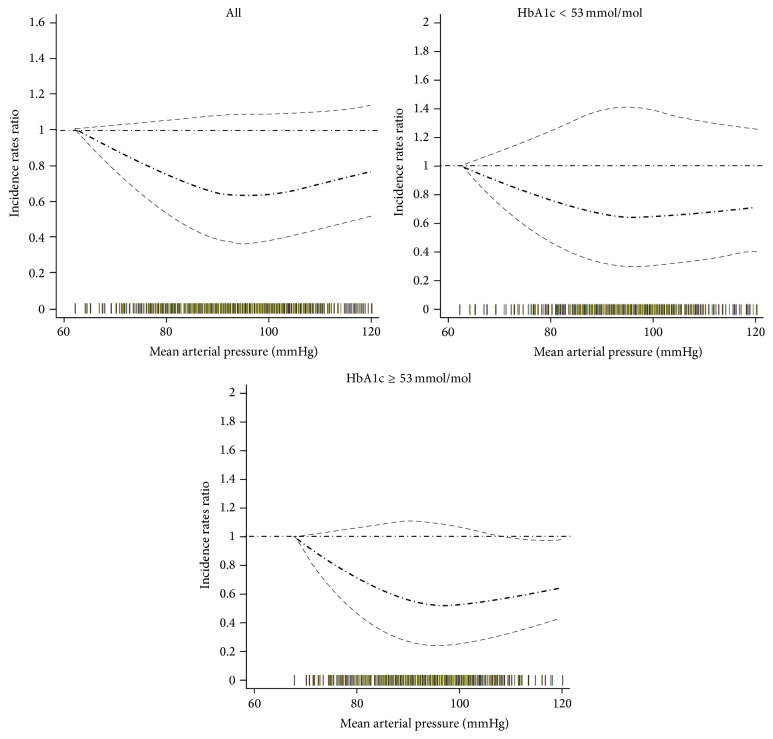
Relationship between mean arterial pressure and cardiovascular hospital admission: analysis in data rich range. Adjusted for prevalent recorded history of cardiovascular/cerebrovascular disease, duration of diabetes registration in GP practices, body mass index, triglyceride, total cholesterol, low density lipoprotein, high density lipoprotein, estimated glomerular filtration rate, smoking status, lipid-lowering treatment, aspirin treatment, and pulse pressure. The thick dash-dot line indicates the incidence rate ratio and the thin dash line indicates the 95% confidence interval. The rag plot (bars on the *x*-axis) presents the mean arterial pressure distribution.

The nonlinear association was also found within the data rich range (60–120 mmHg for MAP) (Figure 2) with significantly higher CVD hospitalisation below/above the 97 mmHg MAP threshold. The interaction between MAP and HbA1c in predicting CVD hospitalisation was similar to the interaction observed in the full data range. Qualitatively similar findings were found for the association between MAP and CVD hospitalisation after excluding cases whose hospital admissions were recorded in the first 3 months of follow-up (see Supplementary Figure 1 and Supplementary Figure 2 in Supplementary Material available online at http://dx.doi.org/10.1155/2016/8714745). In another sensitivity analysis restricting MAP to 80–120 mmHg, a similar association was revealed (Supplementary Figure 3).

Finally, we estimated that any unmeasured confounder that was 1.5-fold as frequent among those with MAP < 97 mmHg as it was among people with MAP outside this range would need to decrease the risk of cardiovascular hospitalisation by a factor of ≥20 to fully explain the results, if no increased risk actually existed (Supplementary Figure 4).

## 4. Discussion

We have shown, to our knowledge for the first time, that MAP and CVD hospitalisation show a nonlinear relationship among patients with type 2 diabetes within a U shaped relationship and a threshold of 97 mmHg. The MAP interaction with HbA1c was particularly of interest, with a lower HbA1c below the MAP threshold and higher HbA1c above the MAP threshold being associated with the greater risk of cardiovascular hospitalisation.

The association between an elevated MAP and both increased CVD and total mortality [2] in the general population is well established. For example, in the Framingham Heart Study, 14 mmHg increase of MAP was associated with a 35% elevation in CVD risk [16]. Our data are in agreement with these previous reports and show that arterial resistance (represented approximately by MAP) also has an adverse effect on the subsequent risk of CVD in those with type 2 diabetes. A positive association between future CVD risk and MAP in patients with type 2 diabetes was shown by Kodama et al. in a meta-analysis [4] with a 9% increased CVD risk for every +10 mmHg increment of MAP. This supports MAP as a good prognostic predictor of CVD risk in people with type 2 diabetes. MAP was suggested to be a strong predictor of CVD even when SBP was controlled to normal, supporting our view that MAP is an independent risk factor rather than a proxy for SBP or DBP.

In the Physicians' Health Study [17], which investigated 22071 US male physicians aged 40 to 84 years, with a follow-up of 10.8 years, a nonlinear association was found between MAP and risk of CVD. The lowest risk of CVD was found at 93–97 mmHg of MAP among men aged <60 years, and increased CVD risk was identified below and above the threshold. However, to our knowledge, the relationship between MAP and hospitalisation, especially CVD hospitalisation, has not been investigated before and might be an indicator for healthcare for people with type 2 diabetes [5, 6]. Ours are consistent with previous findings and provide further evidence of a nonlinear association between MAP and CVD hospitalisation. We have also identified that there is a threshold with the lowest risk of CVD hospitalisation present at 93–101 mmHg of MAP.

Our finding of an interaction between MAP and HbA1c suggests that patients with a low MAP should not have a lower target HbA1c. This is in keeping with the generic guidelines around setting a higher HbA1c target among patients with CVD and particularly followed the findings from the Veterans [18] study that lower HbA1c among patients with prevalent CVD is associated with greater adverse outcomes including death. We tested whether a low HbA1c, among patients with a MAP below threshold, would be amenable to new post hoc studies using existing RCT data.

MAP is thought to act as a surrogate marker for vascular stiffness resulting from a loss of vascular elasticity [19]. Such loss of elasticity arises from alterations in collagen and elastin structure and function which may arise from the increased oxidative stress, carbonyl stress, and advanced glycation end products associated with the relative hyperglycaemia of diabetes and prediabetes [19]. On the other hand, vascular stiffness also increases with advancing age, leading to coronary heart disease. We speculate that this increase in vascular stiffness could explain the abnormal MAP in type 2 diabetes patients and their excess CVD risks [20].

How MAP or arterial stiffness can be reduced is uncertain: current antihypertensive treatment reduces both SBP and DBP and MAP might not change linearly. However, if a high or low MAP is identified (e.g., ≤90 mmHg or ≥100 mmHg MAP to use rounded numbers and address any accuracy issues), lifestyle factors (e.g., diet, physical activity, and smoking) could be discussed in the context of a new finding and therapy could be intensified. Whether such a strategy is beneficial warrants further study, possibly a randomised controlled trial.

Our study has some limitations including the lack of detailed antihypertensive medicine information. However antihypertensive medicine therapy might not distort the relationship between MAP and risk of CVD hospitalisation identified in this study as such treatment is the “norm” for the majority of type 2 diabetes patients. This is likely to be reflected by the normal mean BP observed in both cases and controls in the present analysis. Blood pressure (and therefore MAP) was not measured with epidemiologically robust methods but reflected “real life” blood pressure measurements. This would have likely reduced the chance of finding differences in view of the increased variance unless there was a systematic bias. On the other hand, such routine medical record data might be more readily available for individual risk prediction than other measures. Reverse causality (i.e., the index blood pressure was measured after the episode of illness that led to the hospital admission) is unlikely to have contributed to our findings as there was at least 50 days between the index blood pressure and hospital admission. Naturally, those with eg cardiomyopathy would be expected to be included in the low MAP group and have an increased chance of hospitalisation. The relationship between MAP and CVD hospitalisation might be greater in populations where CVD risk has not been reduced through high lipid-lowering therapy uptake. Indeed, the relatively limited sample size and the potential for our adjustments to be insufficient to address any unknown factors support the testing of these associations and the threshold in a prospective study of longer duration. Although the multicollinearity between MAP and other covariables was not identified in our study, further validation with external data with a large sample size is needed. Further limitations are that only a proportion of GPs in the area provided their data and these were restricted to EMIS users. We have no reason to believe that diabetes care among EMIS users contributing data was any better or worse than other GPs, but this is a limitation. GP diabetes coding could be a limitation [21], but the approach has been used previously and is considered valid.

In summary, nonlinear relationship exists between MAP and cardiovascular hospital admission in type 2 diabetes patients. The MAP threshold for the lowest hospital admission was 97 (95% CI: 93, 101) mmHg. In people with type 2 diabetes, the MAP interacted with HbA1c in defining the risk of cardiovascular hospitalisation. MAP might be a useful measure to consider risk in clinical practice.

## Supplementary Material

Supplemental Figures 1 and 2 show sensitivity analyses of the relationship between mean arterial pressure and cardiovascular hospital admission, excluding admissions in the first 3 month of follow-up with analyses in the full data and data rich range respectively.

## Figures and Tables

**Table 1:** Characteristics of patients with cardiovascular hospitalisation and matched controls.

	Cases (*n* = 588)	Controls (*n* = 2920)	*P* value
Systolic blood pressure, mmHg	135.1 ± 12.9	134.0 ± 11.1	0.0264
Diastolic blood pressure, mmHg	73.0 ± 10.1	72.0 ± 10.0	0.0368
Mean arterial pressure, mmHg	94.1 ± 10.2	91.3 ± 9.2	0.0130
Pulse pressure, mmHg	62.1 ± 12.1	61.0 ± 12.0	0.0165
Body mass index, kg/m^2^	30.4 ± 5.6	29.0 ± 5.8	<0.0001
HbA1c, mmol/mol (%)	62 (7.8) ± 18 (1.6)	57 (7.4) ± 14 (1.3)	<0.0001
Total cholesterol, mmol/L	4.1 ± 1.0	4.2 ± 1.0	0.0270
Triglyceride, mmol/L	2.0 ± 1.0	1.7 ± 0.9	<0.0001
High density lipoprotein, mmol/L	1.1 ± 0.3	1.2 ± 0.4	0.0020
Low density lipoprotein, mmol/L	2.2 ± 0.8	2.4 ± 0.8	0.0060
Estimated glomerular filtration rate, mL/min/1.73 m^2^	56.8 ± 18.9	56.6 ± 17.9	0.865
Current smoker/ex-smoker	66.9	65.8	0.623
Pulse pressure, mmHg	60.9 ± 14.9	61.9 ± 13.8	0.426
Coronary heart disease history, %	66.8	35.6	<0.0001
Cerebrovascular disease, %	16.8	14.4	0.1320
Lipid-lowering treatment, %	86.9	72.2	<0.0001
Aspirin treatment, %	66.9	53.7	<0.0001
