# Supplementary material for: Interaction between Mean Arterial Pressure and HbA1c in Prediction of Cardiovascular Disease Hospitalisation: A Population-Based Case-Control Study
Source: J Diabetes Res. 2016 Jun 13;2016:8714745. doi: 10.1155/2016/8714745 (PMC4921624; doi:10.1155/2016/8714745)
Supplement: Supplementary file 1 — Supplemental Figures 1 and 2 show sensitivity analyses of the relationship between mean arterial pressure and cardiovascular hospital admission, excluding admissions in the first 3 month of follow-up with analyses in the full data and data rich range respectively. [file 8714745.f1.pdf]

Supplemental Figure 1. Sensitivity analysis: relationship curves for association between mean arterial pressure and cardiovascular hospital admission (excluding the admissions in the first 3 month of follow-up): analysis in full data range

Adjusted for age, gender, prevalent recorded history of cardiovascular/cerebrovascular disease, duration of diabetes registration in GP practices, body mass index, triglyceride, total cholesterol, low density lipoprotein, high density lipoprotein, estimated glomerular filtration rate, smoking status, lipid-lowering treatment, aspirin treatment and pulse pressure.

The thick dash-dot line indicates the incidence rate ration and the thin dash line indicates the 95% confidence interval. The rag plot (bars on the x-axis) presents the mean arterial pressure distribution.

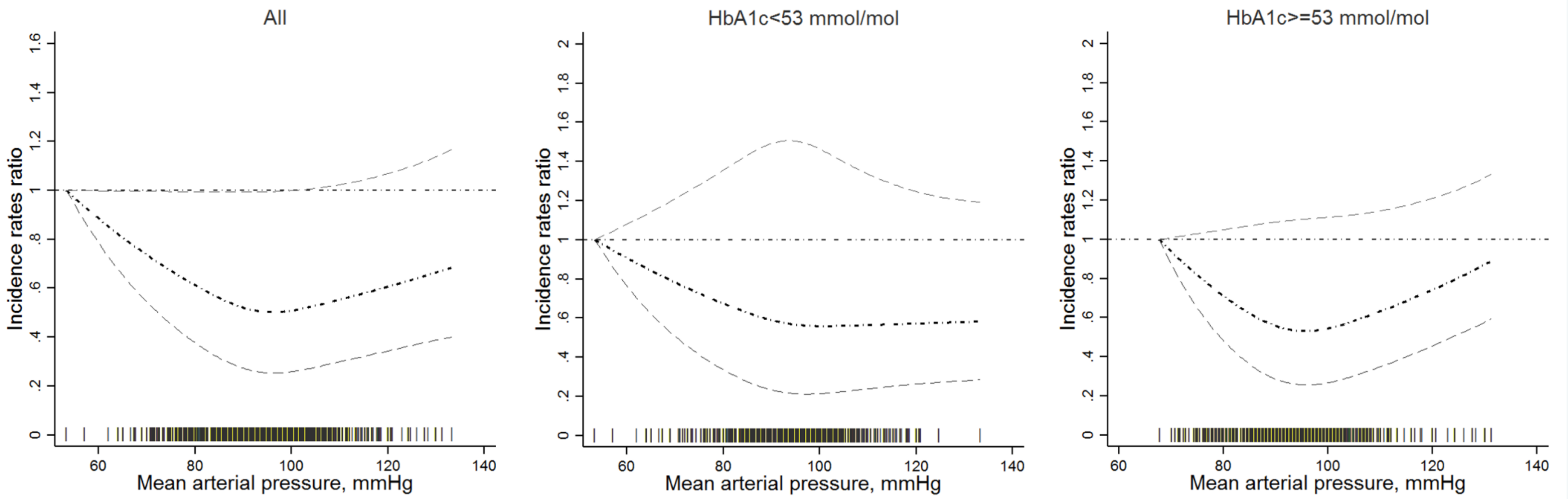

Supplemental Figure 2. Sensitivity analysis: relationship curves for association between mean arterial pressure and cardiovascular hospital admission (excluding the admissions in the first 3 month of follow-up): analysis in data rich range

Adjusted for age, gender, prevalent recorded history of cardiovascular/cerebrovascular disease, duration of diabetes registration in GP practices, body mass index, triglyceride, total cholesterol, low density lipoprotein, high density lipoprotein, estimated glomerular filtration rate, smoking status, lipid-lowering treatment, aspirin treatment and pulse pressue.

The thick dash-dot line indicates the incidence rate ration and the thin dash line indicates the 95% confidence interval. The rag plot (bars on the x-axis) presents the mean arterial pressure distribution.

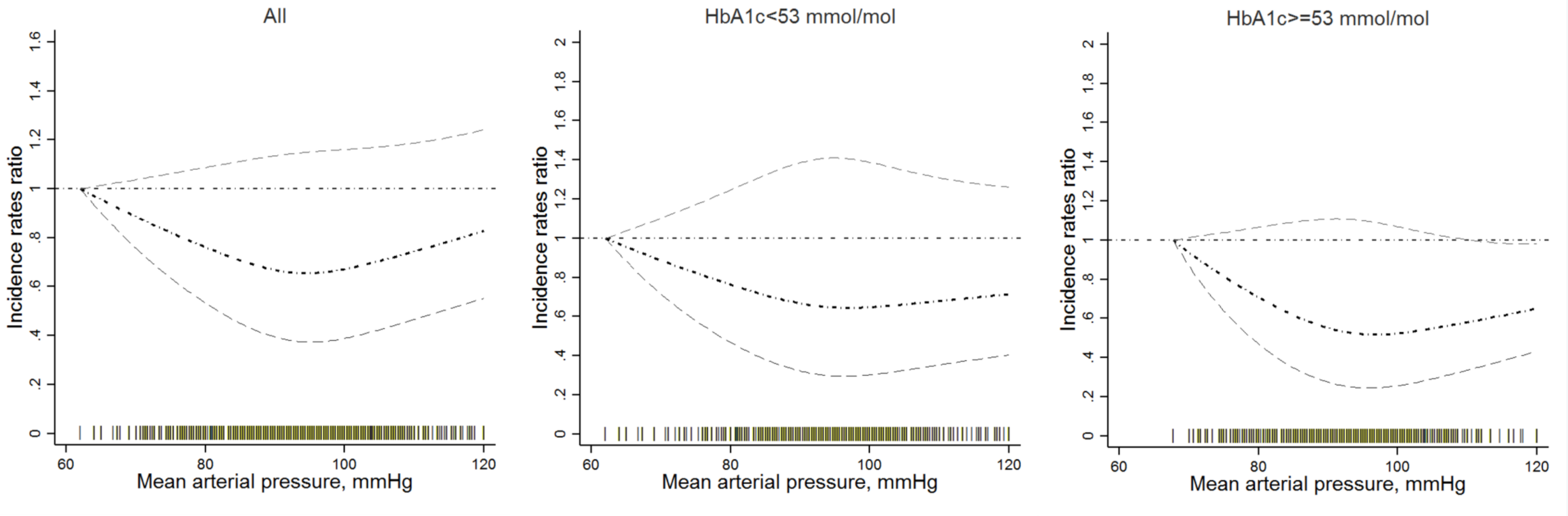

Supplemental Figure 3. Sensitivity analysis: relationship curves for association between mean arterial pressure and cardiovascular hospital admission in MAP of 80-120mmHg

Adjusted for age, gender, prevalent recorded history of cardiovascular/cerebrovascular disease, duration of diabetes registration in GP practices, body mass index, triglyceride, total cholesterol, low density lipoprotein, high density lipoprotein, estimated glomerular filtration rate, smoking status, lipid-lowering treatment, aspirin treatment and pulse pressure.

The thick dash-dot line indicates the incidence rate ration and the thin dash line indicates the 95% confidence interval. The rag plot (bars on the x-axis) presents the mean arterial pressure distribution.

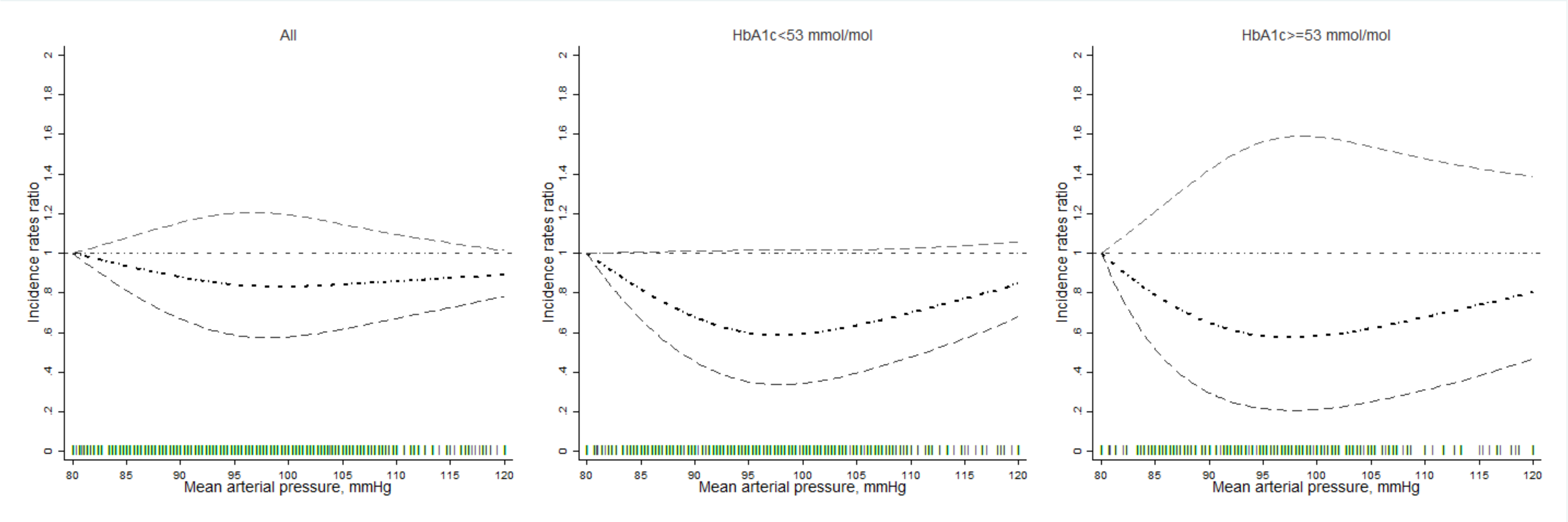

Supplemental Figure 4. Sensitivity analysis: Required Strength of an Unmeasured Confounder

Sensitivity analysis illustrating how strongly an unmeasured confounder would need to be associated with pulse pressure (prevalence ratio for exposure-confounder association, PREC) and cardiovascular hospitalisation (relative risk of the disease in patients with the confounder, RRCD) to fully explain our estimates. We assumed that the prevalence of the confounder was as common as 90% of the population and that 60% of the population with mean arterial pressure less than 97 mmHg. The graphs depict the adjusted incidence rate ratio (IRR) associated with the threshold of pulse pressure (cross line) along with the lower limit of the 95% confidence interval (circle line).

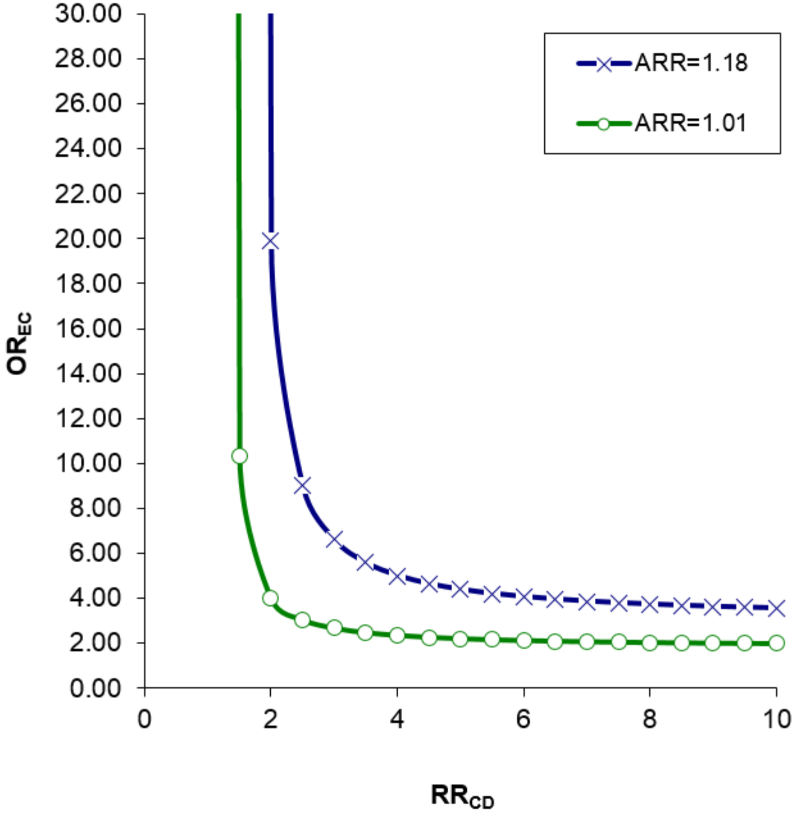

30.00
28.00
26.00
24.00
22.00
20.00
18.00
16.00
14.00
12.00
10.00
8.00
6.00
4.00
2.00
0.00
0
2
4
6
8
10
OR$_{EC}$
RR$_{CD}$
ARR=1.18
ARR=1.01
